# BDNF polymorphisms across the spectrum of psychiatric morbidity: A protocol for a systematic review and meta-analysis

**DOI:** 10.1097/MD.0000000000022875

**Published:** 2020-11-06

**Authors:** Jingzhi Su, Peiqu Liu, Bangshan Liu, Yan Zhang

**Affiliations:** a Department of Psychiatry, The Second Xiangya Hospital; b Department of Anatomy and Neurobiology, School of Basic Medical Science, Central South University; c Mental Health Institute of Central South University, China National Clinical Research Center on Mental Disorders (Xiangya), China National Technology Institute on Mental Disorders, Hunan Technology Institute of Psychiatry, Hunan Key Laboratory of Psychiatry and Mental Health, Changsha, Hunan, China.

**Keywords:** brain-derived neurotrophic factor polymorphisms, meta-analysis, psychiatric morbidities

## Abstract

**Background:**

Nowadays, psychiatric morbidities are more and more common, which imposes huge social and economic burden on all countries across the world. Mental illnesses are found to be related to genetics. Over the past few years, a large number of risk genes and loci related to psychiatric morbidities have been reported. The brain-derived neurotrophic factor (BDNF) is one of the main candidates in neuropsychiatric genetics. However, different studies have shown inconsistencies regarding effect modification of BDNF polymorphisms on psychiatric morbidities. Therefore, in the present study, we aim to qualitatively and quantitatively summarize the relationship between BDNF single nucleotide polymorphisms (SNPs) and various psychiatric morbidities through a meta-analysis.

**Methods:**

PubMed, Web of Science and Embase will be searched using a specified search strategy to identify relevant studies up to April 2020. The meta-analysis will be performed on (1) allele model, (2) dominant model, (3) recessive model, (4) homozygote, and (5) heterozygote model. Sensitivity analyses will be conducted to explore the impact of individual studies on the overall result by evaluating the odd ratios (ORs) with their corresponding 95% confidence intervals (CIs) before and after removing each of the studies from our meta-analysis. Chi-square test will be used to determine whether the observed allele or genotype frequencies in the controls are consistent with HWE. The statistical heterogeneity will be verified by I^2^ statistics. The fixed effects model is needed to estimate the ORs and 95% CIs when there was no heterogeneity between results of included studies (I^2^ < 50%); instead, the random effects model should be used when results of included studies showed significant heterogeneity (I^2^ > 50%). Publication bias will be evaluated with the use of Begg test and Egger test (*P* < .05 is considered statistically significant).

**Discussion:**

With this protocol, a methodology is established that explores the effect modification of BDNF polymorphisms on the association with psychiatric morbidities. Findings from this meta-analysis can provide significant insight into the etiology of psychiatric morbidities.

**Registration:**

Open Science Framework (OSF) Preregistration. September 15, 2020. OSF.IO/QS7XT

## Abstract 2

Supplemental Digital Content is available in the text

## 1 Introduction

In 2017, there were 792 million people living with psychiatric morbidities, which is slightly more than one in ten people worldwide (10.7%).^[1]^ There are more than 200 classified forms of mental disorders, with most common ones being major depressive disorder (MDD), schizophrenia, attention-deficit/hyperactivity disorder (ADHD) and bipolar disorder (BD). Currently, MDD is one of the major causes of global burden of disease, affecting more than 300 million people across the world. Ranking the third most debilitating disorder worldwide at present, MDD is expected to top the list by the year 2030.^[1,2]^ Schizophrenia is still one of the most chronic, debilitating and costly psychiatric morbidities, affecting over 20 million people worldwide.^[1]^ The ever-increasing burden caused by psychiatric morbidities has a significant impact on social and human rights, and may lead to economic consequences in all countries.

The pathological mechanisms of psychiatric morbidities are extremely complex. Despite great progress in research on psychiatric morbidities, our current knowledge about the molecular mechanisms of the development of psychiatric disorders is still very limited.

Some studies have indicated that the brain derived neurotrophic factor (BDNF) is involved in the etiology of the disorders.^[3–6]^ BDNF, belonging to the neurotrophin family of growth factors, is known for the function in the neuronal plasticity in adult brains.^[7]^ By binding to a high affinity p75 neurotrophin receptor and /or tyrosine kinase receptor B (TrkB), BDNF plays an important role in the growth, and differentiation of the developing nervous system. Recently, it is believed that single nucleotide polymorphisms (SNPs) in the BDNF gene is associated with several neuropsychiatric morbidities such as schizophrenia, depression, ADHD and BD.^[8–13]^ One of the most reported, as well as most functional, SNPs in the BDNF gene is rs6265. The rs6265, also known as Val66Met or G196A polymorphism of BDNF, can lead to in a valine-to-methionine substitution at codon residue 66.^[14,15]^ The Met allele is related to less BDNF activity^[16]^ and lower serum levels.^[17]^ It also appears to be associated with memory impairments,^[16,18]^ reduced hippocampal activity.^[19]^ In addition, lower plasma BDNF levels have been reported in depression patients.^[20,21]^ A small amount of the BDNF protein was also found in the post-mortem amygdala, hippocampus, anterior cingulate cortex as well as prefrontal cortex in patients with depression.^[22–24]^

The association between BDNF SNPs and susceptibility to psychiatric disorders has been widely studied, although with inconsistent results. For instance, despite some study results suggesting that the Val66Met variant was not associated with any of ADHD, MDD, schizophrenia,^[10,25–28]^ some studies found that the homozygous carriers Met/Met was increasing risk of schizophrenia compared to the heterozygous state.^[29]^ It also found the Met allele was associated with an increased risk for depression.^[30]^ With the need to combine data from individual studies, meta-analysis, a powerful statistical method and quantitative approach, was adopted to examine the data in a holistic manner and explain the heterogeneity.

The aim of this study is to integrate results from association studies between BDNF SNPs and different psychiatric morbidities, which were further quantified using meta-analysis.

## 2 Methods and analysis

### 2.1 Study registration

This meta-analysis aims to compare the genotype and allele frequencies of BDNF gene polymorphisms in patients with psychiatric disorders (MDD, suicide, mania, BD, UD, schizophrenia and ADHD) and healthy controls, and further to confirm whether BDNF polymorphisms are associated with psychiatric morbidities. This study was registered on the Open Science Framework (OSF) registries (OSF.IO/QS7XT), in accordance with guidelines in Preferred Reporting Items for Systematic Reviews and Meta-Analyses Protocols (PRISMA-P).^[31]^

### 2.2 Eligibility criteria

The inclusion criteria are

(1) patients diagnosed with targeted psychiatric morbidities (major depression disorder, suicide, mania, UD, BD, schizophrenia and ADHD);
(2) observational studies investigating the link between the BDNF polymorphism and targeted psychiatric morbidities;
(3) observational studies on patients with targeted psychiatric morbidities and healthy controls;
(4) studies in which the frequency of alleles and genotype distribution was available;
(5) studies in which the allele and genotype distribution of the control population met the Hardy-Weinberg equilibrium (HWE) model.

### 2.3 Data sources

Systematic literature searches will be performed across platforms or databases such as PubMed, Web of Science and Embase by applying the specified search strategy to identify relevant studies up to April 2020. The references of the retrieved literature will be manually searched, and relevant studies and systematic reviews will be scanned for additional eligible studies.

### 2.4 Search strategy

An extensive systematic search in Embase, PubMed and Web of Science (last updated April 2020) will be conducted, using “brain-derived neurotrophic factor” and the psychiatric diagnoses “depression”, “suicide” “mania”, “unipolar depression”, “bipolar disorder”, “schizophrenia”, “attention-deficit/hyperactivity disorder”, as keywords and Medical Subject Headings. The search strategy is shown in Supplemental Digital Content (Appendix 1). It will appropriately adjust the search terms to conform to different syntax rules of the platforms and databases mentioned above.

### 2.5 Study selection

**Figure 1:**
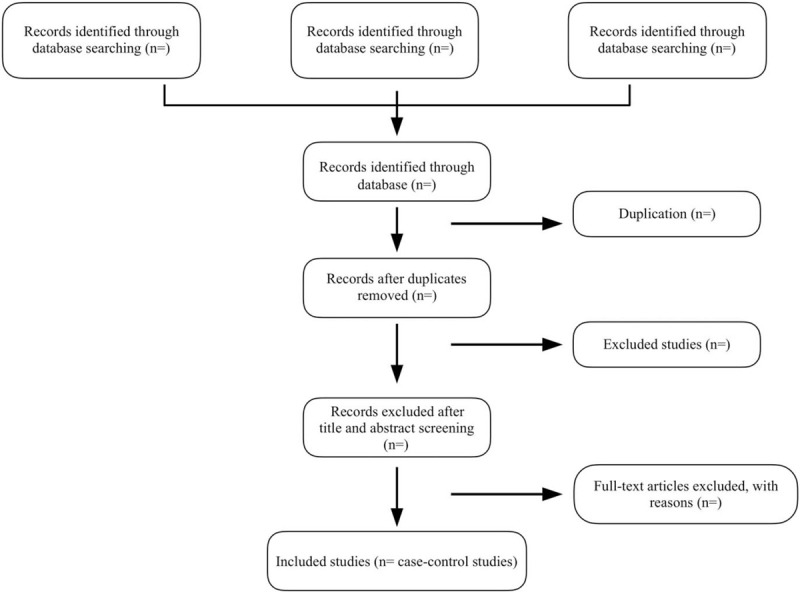
Literature screening process.

The titles and abstracts of all retrieved records will be investigated by 2 authors independently to identify eligible trials. Two reviewers will screen each record retrieved by EndNote independently and go through the full text of all potential literatures for further assessment, in order to exclude irrelevant studies or determine their eligibility. The publication date or language will not be restricted. The detailed selection process will be presented in the flow diagram of Preferred Reporting Items for Systematic Reviews and Meta-Analyses (Fig. 1). Disagreements between the 2 authors, if there is any, will be discussed with a third author.

### 2.6 Data extraction

The two investigating authors will extract the following information with the use of a standardized collection form: publication information (name of author, country of origin, year of publication), information of cases and controls (sample size, age, sex), diagnosis and the diagnostic criteria, and genotype and allele frequency of BDNF SNPs. If any concerned information is not reported in an included study, the missing data will be obtained as much as possible using alternative methods, including contacting the author(s) directly via email.

### 2.7 Evaluation of study quality

The methodological quality of these studies will be evaluated by two independent reviewers independently with the use of modified Newcastle-Ottawa Scale (NOS),^[32]^ which comprises three aspects of the quality: selection, comparability and exposure of cases and controls. The three aspects of quality are selection, comparability and outcome measures for cohort or cross-sectional studies. There are four, two and three items (each account for 1 point) in the three aspect, respectively; thus, a study can be scored a maximum of 9 points if it conforms with all nine items. A study with a score of over 6 is regarded as of high quality.

### 2.8 Statistical analysis

Data will be processed with Stata 15.0 (Stata Corp, College Station, TX). Chi-square test will be used to determine whether the observed allele or genotype frequencies in the controls are consistent with HWE. The statistical heterogeneity will be verified using I^2^ statistics. Fixed effects model will be applied to estimate the odd ratios (ORs) and 95% confidence intervals (CIs) when there were no heterogeneity between results of included studies (I^2^ ≤ 50%); the random effects model will be used when high heterogeneity is shown (I^2^ > 50%).^[33]^ Publication bias will be evaluated using the Begg test^[34]^ and the Egger test^[35]^ (*P* < .05 is considered statistically significant).

Meta-analysis will be performed on the

(1) allele model,
(2) dominant model,
(3) recessive model,
(4) homozygote, and
(5) heterozygote model.

To evaluate demographic region-specific effects or any other effects, we will perform subgroup analysis, dividing subjects into different populations or other categories. Sensitivity analyses will be conducted to detect the impact of individual studies on the overall findings through evaluating the ORs with its corresponding 95% CIs before and after removing each study from the meta-analysis.

### 2.9 Ethics and dissemination

As no private and confidential patient data will be contained in the report, approval from an ethics committee is not required. The results will be published in a peer-reviewed journal. The study raises no ethical issues.

## 3 Discussion

Nowadays, psychiatric morbidities are among the most common health conditions as well as the most common causes of disability across the world.^[1]^ In addition to having serious impact on people's physical health, psychiatric morbidities are also associated with the prevalence, progression, and outcome of some of today's most concerned chronic diseases, including diabetes, heart disease, and cancer.^[36–38]^ The occurrence of mental illnesses is related to genetic factors, which might explain a large part of psychiatric morbidities. Over the past few years, a large number of risk loci related to psychiatric morbidities have been reported.^[39–41]^ These impressive studies have applied the method of genome wide association studies (GWAS) on millions of DNA variants, mainly SNPs, in large population cohorts of hundreds of thousands of individuals.

BDNF is one of the major candidates in neuropsychiatric genetics; many studies have been conducted to detect the possible associations between polymorphisms in the BDNF gene and psychiatric morbidities.^[20,42]^ However, regarding the effect modification of BDNF polymorphisms on the association with psychiatric morbidities, findings from different studies published to date appear to be inconsistent. In view of this, we will use 5 models:

(1) allele model,
(2) dominant model,
(3) recessive model,
(4) homozygote, and
(5) heterozygote model to evaluate the correlation between BDNF polymorphisms and targeted psychiatric morbidities comprehensively.

With the need to combine data from individual studies, meta-analysis, which is a powerful statistical method and quantitative approach, was adopted to examine the data in a holistic manner, as well as examine and explain the heterogeneity. The results of this study will expand the existing knowledge base regarding the pathogenesis of psychiatric morbidities, as well as develop preventive or treatment strategies in this field.

## Author contributions

**Conceptualization:** Yan Zhang

**Data curation:** Jingzhi Su, Peiqu Liu, Bangshan Liu

**Methodology:** Jingzhi Su, Peiqu Liu

**Writing – original draft:** Jingzhi Su

**Writing – review & editing:** Bangshan Liu, Yan Zhang

## Supplementary Material

Supplemental Digital Content
