# Supplementary material for: BDNF polymorphisms across the spectrum of psychiatric morbidity: A protocol for a systematic review and meta-analysis
Source: Medicine (Baltimore). 2020 Nov 6;99(45):e22875. doi: 10.1097/MD.0000000000022875 (PMC7647535; doi:10.1097/MD.0000000000022875)
Supplement: Supplemental Digital Content [file medi-99-e22875-s001.docx]

| **Number** | **Search term** |
| --- | --- |
| #1 | (polymorphism[tiab] or SNP*[tiab] or variant*[tiab] or mutation[tiab] or genotype[tiab] or allele[tiab] or haplogroup[tiab] or haplotype[tiab] or “genetic predisposition”[tiab] or “genetic susceptibility”[tiab] or “Polymorphism”, “Single Nucleotide”[MeSH]) |
| #2 | “BDNF”[tiab] or “Val66Met”[tiab] or “brain derived neurotrophic factor”[tiab] or “ANON2”[tiab] or “BULN2”[tiab] or “[rs6265](https://www.ncbi.nlm.nih.gov/snp/rs6265)”[tiab] |
| #3 | “depression”[tiab] or “depressive disorder*”[tiab] or “major depression”[tiab] or “depressive symptoms”[tiab] or “major depression disorder*”[tiab] or suicide[tiab] or “suicidal behavior”[tiab] or “suicide attempt”[tiab] or “mania”[tiab] or “unipolar depression”[tiab] or “UD”[tiab] or “bipolar disorder”[tiab] or “BD”[tiab] or “schizophrenia”[tiab] or “attention-deficit/hyperactivity disorder” or “ADHD”[tiab] |
| #4 | #1 and #2 and #3 |

Tiab = title/abstract; UD = unipolar depression; ADHD = attention-deficit/hyperactivity disorder

**Appendix 1:** Electronic search strategy in PubMed
